# Preliminary Investigation towards the Use of Infrared Technology for Raw Milk Treatment

**DOI:** 10.3390/foods13071117

**Published:** 2024-04-06

**Authors:** Luigi Danesi, Maria Nobile, Mauro Fontana, Erica Tirloni, Luca Maria Chiesa, Federica Savini, Roberto Edoardo Villa, Sara Panseri

**Affiliations:** 1 Department of Veterinary Medicine and Animal Science, University of Milan, Via dell’Università 6, 26900 Lodi, Italy; luigi.danesi@unimi.it (L.D.); maria.nobile1@unimi.it (M.N.); luca.chiesa@unimi.it (L.M.C.); roberto.villa@unimi.it (R.E.V.); sara.panseri@unimi.it (S.P.); 2 Dirigente Veterinario AULSS9 Scaligera, Via S.M. Crocifissa di Rosa, 37067 Verona, Italy; waxysrlsb@gmail.com; 3 Department of Veterinary Medical Sciences, Faculty of Veterinary Medicine, University of Bologna, Ozzano Emilia, 40064 Bologna, Italy; federica.savini3@unibo.it

**Keywords:** milk, infrared radiation, food safety, sustainability, green transition

## Abstract

Infrared (IR) technology offers a promising solution for reducing microbiological loads in various food types while preserving their quality traits, such as flavour. However, research on IR’s application in complex matrices is limited. Therefore, our preliminary study aimed to evaluate its effectiveness in sanitizing bovine raw milk. We assessed the bacterial count before and after IR treatment by comparing volatile organic compound profiles via headspace extraction and GC/MS analysis. Our findings showed that higher energy levels led to a greater bacterial reduction. IR85 was the most effective in reducing *Coliforms* and *Enterobacteriaceae* in non-homogenised samples, with a reduction ranging from −1.01 to >−2.99 and from −1.66 to −3.09 Log CFU/mL, respectively. IR60 and 70 showed no efficacy, while IR80 had intermediate but still satisfactory effect. IR85 notably affected volatile compounds, particularly increasing hexanal (from 0.08 to 4.21 ng g^−1^) and dimethyl sulphone (from 10.76 to 26.40 ng g^−1^), while IR80 better preserved the aroma profile. As a result, only IR80 was tested with homogenised raw milk, demonstrating significant bacterial reduction (from >2.39 to 3.06 Log CFU/mL for *Coliforms* and from 1.90 to >2.45 Log CFU/mL for *Enterobacteriaceae*) and maintaining the aroma profile quality.

## 1. Introduction

According to the Sustainable Development Goals promoted by the United Nations within the 2030 agenda, one of the most significant challenges in the coming years is promoting a safe and environmentally friendly food system for consumers [1]. The world’s growing population will demand more food, but the simultaneous reduction in natural resources and increased attention to environmental issues will make it harder to meet that demand [2]. Therefore, finding new technologies and techniques to reduce the environmental impact while maintaining high levels of safety and productive efficiency is pivotal. Water consumption, for example, is a key aspect to consider; milk processing is one of the most water-intensive industries in the agro-food system.

High amounts of water are used for production needs as raw material or cooling machinery and washing equipment [3]. Water conservation and protection are crucial since it is a valuable resource, as emphasized by the sixth point of the Sustainable Development Goals [1]. In this context, the economic and energy costs, especially for milk sanitization, also play a crucial role in the sustainable transition of dairy industries today.

Milk and dairy consumption have a rich historical tradition in Western and European countries, where milk consumption remains one of the highest in the world (EU-27 per capita milk consumption of 53.28 kg in 2022) [4]. Demand for fresh milk is expected to increase in the coming decades, driven principally by countries such as India and Pakistan, while in Europe and North America per capita demand for fresh dairy products is stable to declining [5]. In addition, cheese consumption is expected to grow in emerging countries as well as in Europe and North America [5]. Milk is a highly versatile food, as it can be consumed directly; processed to produce derivatives such as cheese, butter, and yogurt; or used as an ingredient in industrial preparations [6]. In recent years, raw milk, believed to be of superior quality to milk that has undergone heat treatment (pasteurisation and sterilization), has regained popularity. However, the consumption of raw milk can pose a potential risk from a microbiological point of view [7,8], primarily due to the presence of various types of microorganisms such as bacteria, viruses, fungi, protozoa, and yeasts [9]. Milk products are typically subjected to heat-based treatments to ensure their safety. Pasteurisation is a common method, and its efficacy is based on the relationship between temperature and time of treatment. According to current European regulations (Reg EC 2074/2005), pasteurisation procedures must follow either high temperature for a brief period (minimum 72 °C for 15 s), low temperature for an extended period (minimum 63 °C for 30 min), or any alternative combinations of time and temperature that produce equivalent results [10]. Although heat treatments are crucial for ensuring consumer safety, they present various challenges and critical aspects. Pasteurisers burn a large amount of fossil fuels, making them unsustainable from an environmental perspective [11]. Additionally, thermal treatments can negatively affect the quality of the final product, altering its physical, sensory, and nutritional properties [12,13,14,15]. Volatile compounds are particularly susceptible to alteration during heat treatment, which is relevant considering their crucial role in food product evaluation. Previous studies have shown that raw milk cheeses usually have stronger flavours and higher concentrations of volatile compounds compared to pasteurised ones [16]. This difference is often recognised by consumers. As reported by Ratschi et al., 2021 [17], who compared two different productions of the same cheese, one made from raw milk and the other from pasteurised, when asked to express a preference, the panel of tasters indicated a higher acceptability for the product made from raw milk.

Several technologies have been evaluated in recent years as potential alternatives to common heat-based methods. These technologies include Infrared (IR) heating, Ultraviolet Light, Cold Plasma, Pulse Electric Fields, Radio Frequency Heating, Microwave Heating, High-Pressure Processing, and Ohmic Heating [15,18,19]. Concerning IR, it is a part of the electromagnetic spectrum between the visible region and microwaves, with a wavelength from 0.5 to 1000 μm. Particularly, it is categorized into near-IR (NIR) ranging from 0.75 to 1.4 μm, mid-IR (MIR) from 1.4 to 3μm, and far-IR (FIR) from 3 to 1000 μm [20]. FIR radiation is well-suited for food processing since most food components absorb radiative energy within this range. IR penetrates substances, causing water molecules to vibrate and produce heat. This vibration frequency ranges from 60,000 to 150,000 MHz. The radiant energy dissipates as heat, warming the food surface. The penetration depth depends on the product’s thickness, water activity, components, and the wavelength of the Infrared radiation [20]. Among the different technologies, IR stands out for its numerous benefits, such as high heat transfer rates, uniform heating, less water usage, the possibility to be combined with traditional convection heating methods, and low heating time [18]. These characteristics of IR technology make it an attractive option potentially adoptable in marginal areas such as mountains, contexts where electricity and water access are very limited and usually more expensive [21]. These areas are recognized as stock of the world’s biodiversity, but unfavourable economic conditions often lead to their abandonment, resulting in a loss of characteristic productions and competitiveness of local farms as a result [22]. IR is already adopted in the food industry for dehydration, heating, flour, roasting, baking, and thawing [23]. Otherwise, multiple studies have investigated IR technology due to its capacity to decrease the presence of various microorganisms in diverse food matrices [24,25,26,27]. Scarce literature is available regarding the application of IR technology for milk sanitation. Krishnamurthy et al., 2008, explored the use of IR as a viable method for milk sanitization [28]. This study investigated the effectiveness of IR toward the reduction of *Staphylococcus aureus* in milk samples. Considering the above-mentioned considerations, the present research aimed to explore the feasibility use of IR technology to treat raw milk investigating its impact on the bacterial species mainly involved in milk spoilage as well as on the volatilome profile (volatile organic compounds—VOCs). Homogenisation, which reduces the particle size of fluids, such as milk, can improve consistency and stability as well as increase the surface area available for IR treatment [29]. Therefore, the most promising treatment was tested on pre-homogenised samples due to its industrial application.

## 2. Materials and Methods

### 2.1. Sample Collection

Overall, five trials based on weekly sampling, were considered in the present study as presented in Table 1. The raw milk was provided by an Italian dairy industry producing different dairy products (pasteurised and UHT milk, butter, and PDO Grana Padano cheese). The milk (60 L for each batch) was sampled directly from the plant tank (100.000 L capacity, refrigerated at 4 °C) connected to the conventional pasteuriser to simulate the actual process while also decreasing the variability at a minimum level in term of the animal trait and feeding system. Milk was transported to the laboratory and maintained at 4 °C until the IR treatment. Trials 1, 2, and 3 involved independent repetitions conducted using three distinct batches of raw milk, all treated under identical conditions. Similarly, trials 4 and 5 were conducted using two different batches. The initial three trials aimed to assess the efficacy of various IR energies (60, 70, 80, and 85) in terms of microbiological and volatilome profiles. Additionally, trials 4 and 5 involved homogenisation, a method recently adopted by some dairy industries to enhance pasteurization efficacy, as a preliminary step before IR treatment. Among the different IR energies tested, only 80, which demonstrated the best results in terms of microbiological reduction and aroma preservation, was utilized in trials involving homogenised milk.

### 2.2. Milk Homogenisation

Milk homogenisation was achieved by using an NS1001L2K benchtop homogeniser (Niro Soavi S.p.A; Parma, Italy). The system was equipped with a barometer to monitor the work pressure; a value between 150–250 bar was maintained during the homogenisation process.

### 2.3. IR Treatment

An IR prototype instrument based on patented technology was used in the present research (N. 102020000007867). The system is characterized by 3 tubular quartz ducts (8 mm i.d. and 1250 mm length). A single emitter with a maximum power of 7000 W at 400 V radiates over 1100 mm was installed (Infrared S.r.l; Rho, Italy). An external panel allowed adjusting the emitter power (Infrared S.r.l; Rho, Italy). The energies applied (60, 70, 80, and 85) indicate the percentage of installed power used at that moment from the total of 7000 W. An external pump charges milk into the ducts (flow rate of 1.5 L/min) and, upon entry, the milk envelops the entire path within the quartz tube, exposing it to Infrared radiation. Positioned near the duct and aligned with the milk flow direction, the IR source usually achieves a temperature of 800 °C. The IR system was configured to emit IR radiation with a wavelength in the 3–5 µm range (FIR) (Infrared S.r.l; Rho, Italy).

### 2.4. Microbiological Analyses

Hygiene parameters were quantified via spread plating using the following indicator bacteria and procedure: The total viable count (TVC) was enumerated onto Plate Count Agar (Scharlab, Barcelona, Spain) and subsequently incubated for 48 h at 30 °C. *Enterobacteriaceae* were enumerated on Violet Red Bile Dextrose Aar (VRBD) (Scharlab, Barcelona, Spain) and then incubated at 37 °C for 24 h. *Coliforms* were enumerated onto Chromocult Agar (Merck KGaA, Darmstadt, Germany) and then incubated at 37 °C for 24 h. Lactic acid bacteria (LAB) were enumerated on De Man Rogosa Sharpe Agar (MRS) and then incubated in anaerobiosis for 48 h at 30 °C. The results were expressed as log CFU/mL.

### 2.5. Volatilome Profiling of Milk

#### 2.5.1. Extraction of Volatile Compounds (VOCs)

Headspace solid-phase microextraction (HS-SPME) was used to investigate the influence of different IR energies on the volatile compounds of milk samples according to Panseri et al., 2011 [30]. Briefly, 10 mL of milk was put into a 20 mL glass vial equipped with a silicon–polytetrafluoroethylene septum in cap (Supelco, Bellefonte, PA, USA); 100 µL of 4-methyl-2-pentanone solution in water at a concentration of 20 µL mL^−1^, was used as an internal standard. To avoid matrix alterations, a temperature of 10 °C was selected both for the extraction and equilibration phases. After the sample equilibration time of 1 h, a conditioned (1.5 h at 280 °C) 85 µm carboxen/polydimethylsiloxane (CAR/PDMS) StableFlex fibre (Supelco; Bellefonte, PA, USA) was exposed to the headspace of the sample for extraction (3 h) using a CombiPAL system injector autosampler (CTC Analytics, Zwingen, Switzerland). During the analysis, vials were placed on a cooling plate to keep the temperature constant (CTC Analytics, Zwingen, Switzerland).

#### 2.5.2. Gas Chromatography–Mass Spectrometry 

A Trace GC Ultra (Thermo-Fisher Scientific; Waltham, MA, USA) Gas Chromatograph coupled to a quadrupole Mass Spectrometer Trace DSQ (Thermo-Fisher Scientific; Waltham, MA, USA) and equipped with an Rtx-Wax column (30 m; 0.25 mm i.d.; 0.25 µm film thickness, Restek, Stockbridge, GA, USA) was employed to perform HS-SPME analysis. The program for the oven temperature was set starting from 35 °C, kept for 8 min, and a secondary increase of 4 °C min^−1^ until the temperature of 60 °C. From 60 °C to 160 °C the temperature increased at a rate of 6 °C min^−1^, while for the last range, from 160 °C to 200 °C, it increased at a rate of 20 °C min^−1^. The fibre was thermally desorbed, after each analysis, in the GC injector at 250 °C for 5 min, to prevent carryover or contamination. The injections were performed in splitless mode (5 min). Helium, at a constant flow of 1 mL min^−1^ was used as a carrier gas. The line for transfer to the mass spectrometer was kept at 230 °C, while the temperature of the ion source was set at 250 °C. Mass spectra were obtained using an electron impact mass selective detector at 70 eV, a multiplication voltage of 1456 V, and data collection at a rate of 1 scan s^−1^ over the m/z range 30–350. Compounds were identified by comparing the retention times of chromatographic peaks with those of authentic compounds tested under the same conditions, when available, or by comparing Kovats retention indices with the literature data. The identification of patterns of MS fragmentation was performed via comparison with those of pure compounds or using the database of the National Institute of Standards and Technology (NIST). Quantitative evaluation was performed using the internal standard procedure, assuming a response factor of one. The quantity results (ng g^−1^) of each volatile compound were then calculated based on the relation of the intensity of the volatile compounds peaks with the intensity of the internal standard added to the sample in a known amount and expressed as ng g^−1^ internal standard equivalents.

### 2.6. Statistical Analysis

The statistical analysis was performed using GraphPad InStat software (version 3.10, GraphPad Software, Inc., La Jolla, CA, USA). Each sample, treated with IR, was compared to the corresponding raw milk. A paired *t*-test was performed to compare the values if they passed the normality test; otherwise, the Wilcoxon matched pairs test was used. The significant difference was set at *p* < 0.05.

## 3. Results and Discussion

### 3.1. Microbiological Analyses

In the present study, five preliminary trials were conducted with the aim to evaluate the effect of IR technology on the microbiological aspects of homogenised and non-homogenised raw milk. Different bacterial groups play a role in milk contamination, with a repercussion on quality, safety, and even beneficial effects. In this study, some important bacterial groups were considered as indicators of the hygienic procedures and conditions during production and may be considered as significant spoilage agents like the mesophilic total aerobic viable count, Enterobacteriaceae, and Coliforms. Coliforms are very important indicators of the sanitary quality of milk and are extremely relevant for the good success of the cheese-making process and aging to obtain high quality finished cheeses. Finally, we also took into consideration a group of bacteria with potential beneficial features such as lactic acid bacteria. European Regulation EC 2073/2005 does not cover any criteria for hygiene indicators specifically applicable for raw milk [31]. Considering the first three trials conducted on non-homogenised milk, in raw milk, TVC was always close to 5 Log CFU/mL; these data agreed with those reported recently by Böhnlein et al., 2021, who found TVC higher than 5 Log CFU/mL in 36.4% of the milk samples collected at farm level in northern Germany [32]. In the same study, Enterobacteriaceae were detected at mean values of 2.7 ± 1.2 Log CFU/mL. In our study, in non-homogenised raw milk, in trial 3 these bacterial group reach up to 4.09 ± 0.12. The application of IR technology allowed us to obtain a decrease in TVC counts (Table 2): this decrease was a function of the energy applied. In fact, with IR 60 and IR 70, no decrease was evidenced in any of the three trials conducted. The effect of the technology was evidenced starting from IR 80: in this case, a limited decrease was detected (from −0.84 to −1.96 Log CFU/mL in the three trials with non-homogenised milk), while when the energy was higher (IR 85) this decrease also reached −2.14 Log CFU/mL. The same trend was also revealed for the other hygiene indicators such as Enterobacteriaceae with substantially no decrease or a very limited decrease using IR 60 and IR 70, while a decrease from −0.73 to −2.44 and from −1.66 to −3.09 occurred using IR 80 and 85, respectively. Also, Coliforms showed very a similar behaviour with no decrease or a very limited decrease using IR 60 and IR 70, while a decrease from −0.47 to −2.54 and from −1.01 to >−2.99 Log CFU/mL occurred using IR 80 and 85, respectively. LAB resulted to be more difficult in terms of the efficacy of the technology applied, with two out of the three trials conducted in non-homogenised milk using IR 80 where the decrease was negligible and equal to −0.36 Log CFU/mL. Applying IR 85, the effect was clearly higher with a decrease from −0.85 to −2.35 Log CFU/mL.

In homogenised raw milk, a decrease in TVC counts was also detected: a decrease was detected using IR 80 (from −0.70 to −1.05 Log CFU/mL in the two trials). The same trend was also revealed for the other hygiene indicators such as Enterobacteriaceae with a decrease from −1.90 to >−2.45. Also, Coliforms showed a very similar behaviour with a decrease from >−2.39 to −3.06 Log CFU/mL. LAB resulted, again, to be more difficult in terms of the efficacy of the technology applied, with a very variable decrease in the two trials (from −0.36 Log to −2.22 Log CFU/mL). Infrared technology has long been underestimated in the food industry, regardless of its great potential. It is generally applied for the dehydration of vegetables, fish, pasta, and rice; for heating flour; and for roasting cereal, coffee, and cocoa. Very few scientific papers have focused on the effect of IR on milk bacterial counts. Only Krishnamurthy et al. (2008) demonstrated an important inactivation of *Staphylococcus aureus* with reduction a from 0.10 to 8.41 Log CFU/mL [28].

### 3.2. Volatilome Profiling of Milk

Flavour represents one of the most important attributes for consumer acceptance of milk [33]. Milk flavour profiles derive from the proportion of different volatile compounds belonging to different chemical classes (ketones, aldehydes, sulphur compounds, alcohols, carboxylic acids, etc.). Its composition is influenced by several factors: animal diet or metabolism; microbial/enzymatic activities; as well as technological processes as pasteurisation, sterilization, and ultra-high temperature (UHT) [34,35,36]. Heat processing generally causes the degradation of the major milk constituents (proteins, sugars, and lipids), resulting in flavour implications, characterized mainly by cooked, scorched, and caramelized sensory notes of the product [37,38]. Tables S1 and S2 showed all the volatile compounds identified through HS-SPME and GC/MS analysis. Table 3 and Table 4 show the volatile compounds typically associated with alterations and off-flavours derived from heat treatment and belonging to the aldehydes, ketones, sulphur compounds, and furans as chemical classes.

In trials 1, 2, and 3, a total of twenty-five molecules were identified, divided into one aldehyde, six ketones, three sulphur compounds, five carboxylic acids, six alcohols, one ether, two hydrocarbons, and one amide (Table S1). In Table S2, the complete VOCs profile for trials 4 and 5, composed of a total of 51 different compounds, are reported: eight aldehydes, eleven ketones, four sulphur compounds, twelve carboxylic acids, seven alcohols, three furans, and six esters. Meanwhile, in Table 3 and Table 4, the profiles of aldehydes, ketones, sulphur compounds, and (when detected) furans are reported. Aldehydes are an essential class of compounds that have a strong impact on the flavour of milk, even at low concentrations [39]. Our results have confirmed that reduced concentrations of aldehydes are generally found in raw milk. Specifically, in non-homogenised milk, only hexanal was identified. It was detected in the raw sample, IR70, IR80, and IR85 at concentrations of 0.08, 0.32, 2.90, and 4.21 ng g^−1^, respectively (Table 3). Hexanal concentration can be increased through thermal treatments due to the phenomena of hydroperoxides decomposition and unsaturated fatty acids autoxidation, both of which are promoted by heat [40]. Our results showed a clear trend in the growth of this compound with increasing applied energy in our samples. While the IR85 sample had a significantly higher content than the other samples, it was still below the odour threshold identified in the literature for this compound, which is 4.5 ng g^−1^ [41]. In trials 4 and 5, greater variability was observed with the presence of 3-methylbutanal, pentanal, acetaldehyde, heptanal, nonanal, furfural, benzaldehyde, as well as hexanal, which, even in homogenised milk, was confirmed as the most abundant aldehyde (5.32 and 8.40 ng g^−1^ in raw and IR80, respectively). An increase in total aldehyde content was noted following treatment, with heptanal and nonanal being among the compounds exhibiting the greatest increase (0.66 and 0.55 ng g^−1^ in IR80 milk). These compounds are particularly sensitive to heat treatment [42]. However, in both cases, the new values obtained were below the odour threshold (3 and 1 ng g^−1^). In heat-treated milk, a stale smell is one of the most unpleasant notes. Methyl ketones are the main culprits for this aromatic note, while cyclic ketones and diketones are responsible for the reheated flavour perceived in UHT milk [43]. Our results, in line with the literature, suggest that 2-propanone is the most abundant compound detected [44]. The production of methyl ketones is related to the β-oxidation degradation of saturated fatty acids [45]. These ketones could have different origins, such as the metabolic reactions in cows for lower molecular weight methyl ketones like 2-propanone and 2-butanone [46]. However, their increase in milk could also be associated with thermal treatments [47,48]. In trials 1, 2, and 3, 2-butanone was present at high concentrations, but no alteration was detected (unlike 2-propanone, which increased in IR70, IR80, and IR85). Similar to aldehydes, a higher variety of methyl ketones were detected in trials 4 and 5. Other than 2-butanone and 2-heptanone, 2-pentanone, 2-octanone, and 2-nonanone were also detected. Comparing the raw milk with the IR80 sample, 2-heptanone and 2-nonanone showed a significant increase, but, in both cases, it was below the odour threshold reported in the literature, which is set at 5 ng g^−1^ [41]. Sulphur compounds are often associated with thermal processes and contribute to the “cooked” flavour of milk that has been heat-treated. These compounds are known to cause off-flavours in milk, as reported by Panseri et al. in 2009 [49]. The high temperatures required during the processing of UHT milk can easily lead to the development of cooked notes, due to the denaturation of whey proteins and the subsequent release of sulphydryl groups [34]. There are several compounds in this category, such as methanethiol, dimethyl disulphide, dimethyl sulphoxide, and dimethyl sulphone. However, their presence in milk may vary, and their relevance to milk flavour may differ. Dimethyl sulphide is a compound that, present in low concentrations (5–10 ng g^−1^), contributes to the pleasant aroma of milk [50]. However, heat treatments can cause its concentration to increase. Our milk samples exhibited the presence of dimethyl sulphide in all samples above 10 ng g^−1^, even in raw milk, which could be attributed to the diet of the cows [51]. Interestingly, non-homogenised milk samples showed a higher content of this compound compared to homogenised ones. In trials 1–3, the average content of dimethyl sulphide in raw milk was 48.37 ng g^−1^, whereas, in trials 4–5, it averaged 21.09 ng g^−1^. This discrepancy may be related to the oxidation of dimethyl sulphide to dimethyl sulphone, which is notably higher in homogenised milk samples. Additionally, the presence of dimethyl sulphoxide, an intermediate in this reaction, might confirm this theory [52]. The results of tests 1–3 indicate a significant increase in the dimethyl sulphide content for all energy levels tested and a significant increase only at energy level 85 for dimethyl sulphone. Conversely, in tests 4–5, there is a statistically significant reduction in dimethyl sulphide, accompanied by an increase in dimethyl sulphone. This suggests that homogenisation and Infrared radiation may somehow promote the oxidation of dimethyl sulphide into dimethyl sulphone. In any case, the presence of dimethyl sulphone is of no concern as it is mostly flavourless [53]. Meanwhile, dimethyl sulfoxide concentrations did not statistically increase in the IR80 sample of homogenised milk samples. Dimethyl disulphide is another relevant compound. Like dimethyl sulphide, it can be produced because of the degradation of methionine in the Strecker reaction. In our study, dimethyl disulphide was not detected in raw milk, in contrast to the IR-treated samples where it maintained a low concentration (highest value of 0.33 ng g^−1^) and was well below the odour threshold for this compound in milk (19 ng g^−1^) [54]. Furans are a class of compounds that are produced when lysine’s ε-amino group reacts with reducing sugar in a process known as the Maillard reaction [55]. These compounds are a potential carcinogen that can be found in heat-treated foods, such as milk beverages [56]. In our study, furans were detected in tests 4 and 5. However, their presence was minimal, and we did not observe any statistically significant differences compared to the corresponding raw milk.

## 4. Conclusions

The application of IR for raw milk decontamination emerges as a promising technology in the dairy industry. This preliminary study demonstrates that IR can effectively reduce bacterial loads without significantly altering the aroma of the treated matrix. The findings of this study underscore the potential of IR technology to revolutionize the conventional methods of raw milk sanitation, promoting a paradigm shift towards more sustainable, efficient, and high-quality dairy processing practices. This research contributes valuable insights into the dairy industry and sets the stage for further exploration, considering the lack of research present in the literature (based on both the safety and quality of treated raw milk). However, it is important to consider the amount of energy used. In the first three trials, energy level 85 showed the best performance in terms of bacterial reduction (Table 2). On the other hand, the use of excessively high energy levels can affect the composition of volatile compounds in the treated milk, causing a rise in compounds, as reported in Table 3. The use of suitable energy, such as IR80, showed promising results in terms of both bacterial reduction and preservation of aromatic characteristics in the two types of milk analysed (homogenised and non-homogenised). The findings of this study suggest several avenues for refinement and enhancement in future applications. These preliminary results could be used as a guide for further experimentation and adjustments, leading to an improvement and industrial-scale development of the process.

## Figures and Tables

**Table 1:** Milk treatment conditions overview.

Trial	Matrix	Energy Tested	Analysis
Trial 1	Raw non-homogenised milk	60, 70, 80, 85	Microbiology and VOCs
Trial 2	Raw non-homogenised milk	60, 70, 80, 85	Microbiology and VOCs
Trial 3	Raw non-homogenised milk	60, 70, 80, 85	Microbiology and VOCs
Trial 4	Raw homogenised milk	80	Microbiology and VOCs
Trial 5	Raw homogenised milk	80	Microbiology and VOCs

**Table 2:** Observed mean reduction in bacteria in raw milk after IR treatment.

Trial	Mean (Log CFU/mL)	TVC	COLIFORMS	ENTEROBAC- TERIACEA	LAB
**1**	Raw milk	4.87	2.31	2.66	3.84
	IR 60	4.70	2.65	3.32	3.81
	Δ	**−0.17**	**0.34**	**0.66**	**−0.03**
	IR 70	4.75	1.36	3.39	3.77
	Δ	**−0.12**	**−0.95**	**0.73**	**−0.07**
	IR 80	3.76	1.57	1.93	3.16
	Δ	**−1.11**	**−0.74**	**−0.73**	**−0.68**
	IR 85	3.30	1.30	1.00	2.84
	Δ	**−1.57**	**−1.01**	**−1.66**	**−1.00**
**2**	Raw milk	5.20	3.99	4.09	4.54
	IR 60	5.37	3.80	4.28	4.95
	Δ	**0.17**	**−0.19**	**0.19**	**0.41**
	IR 70	4.85	3.58	4.11	4.21
	Δ	**−0.35**	**−0.41**	**0.02**	**−0.33**
	IR 80	3.24	1.45	1.65	2.77
	Δ	**−1.96**	**−2.54**	**−2.44**	**−1.77**
	IR 85	3.06	<1.00	1.00	2.19
	Δ	**−2.14**	**>−2.99**	**−3.09**	**−2.35**
**3**	Raw milk	4.98	3.45	3.95	4.30
	IR 60	4.58	3.38	3.58	4.23
	Δ	**−0.30**	**−0.07**	**−0.37**	**−0.07**
	IR 70	4.92	3.00	3.58	4.30
	Δ	**−0.06**	**−0.45**	**−0.37**	**0.00**
	IR 80	4.14	2.98	3.08	3.94
	Δ	**−0.84**	**−0.47**	**−0.87**	**−0.36**
	IR 85	3.52	<1.00	1.30	3.45
	Δ	**−1.46**	**>−2.45**	**−2.65**	**−0.85**
**4**	Raw milk	5.00	4.54	3.40	4.57
	IR 80	4.30	1.48	1.50	4.21
	Δ	**−0.70**	**−3.06**	**−1.90**	**−0.36**
**5**	Raw milk	5.42	3.39	3.45	5.27
	IR 80	4.37	<1.00	<1.00	3.05
	Δ	**−1.05**	**>−2.39**	**>−2.45**	**−2.22**

**Table 3:** VOCs profile of raw non-homogenised milk before and after IR treatment using different energies.

Rt	Compound	Raw Milk (*n* = 3)	S.D. (±)	IR60 (*n* = 3)	S.D. (±)	IR70 (*n* = 3)	S.D. (±)	IR80 (*n* = 3)	S.D. (±)	IR85 (*n* = 3)	S.D. (±)
*Aldehydes*											
8.98	Hexanal	0.08	0.16	n.d.	-	0.32	0.63	2.90	1.39	4.21 *	1.63
**Total**		**0.08**		**n.d.**		**0.32**		**2.90**		4.21 *	
*Ketones*											
2.26	2-Propanone	1212.95	268.77	1405.73	328.22	1369.23 *	253.92	1553.42 *	472.97	1613.35 *	583.46
3.12	2-Butanone	282.75	47.46	268.61	103.14	263.53	67.60	284.33	99.82	282.95	86.80
13.01	2-Heptanone, 6-methyl-	0.26	0.29	0.55	0.29	0.89	0.27	2.93 *	2.00	5.01 *	3.61
13.83	2-Heptanone	0.49	0.33	0.80	0.80	0.53	0.37	0.33	0.12	0.37	0.22
14.82	Propanone, 1,1-dichloro-	0.19	0.19	0.21	0.11	0.38	0.21	1.05 *	0.80	1.77 *	1.29
17.67	3-Hydroxy-2-butanone	1.46	1.84	2.08 *	2.81	2.02 *	2.43	1.06	0.84	0.74	0.24
**Total**		**1498.11**		1677.98 *		1636.58 *		1843.13 *		1904.19 *	
*Sulphur compounds*											
1.90	Dimethyl sulphide	48.37	15.09	58.57 *	25.59	57.37 *	20.13	57.78 *	19.86	58.76 *	20.86
8.32	Dimethyl disulphide	n.d.	-	0.23	0.27	0.05	0.09	0.33	0.46	0.12	0.11
31.05	Dimethyl sulphone	10.76	6.48	10.08	5.06	14.79	2.94	13.00	4.15	26.40 *	11.63
**Total**		**59.13**		68.88 *		72.21 *		71.11 *		85.28 *	

Data expressed as ng g^−1^ SI equivalents; * indicates a significant statistical difference compared to raw milk (*p* < 0.05); n.d., not detected.

**Table 4:** VOC profile of raw homogenised milk before and after IR treatment using energy 80.

Rt	Compound	Raw Milk (*n* = 2)	S.D. (±)	IR80 (*n* = 2)	S.D. (±)
*Aldehydes*					
3.39	3-Methyl-butanal	1.02	1.09	0.63	0.83
4.74	Pentanal	0.23	0.18	0.37	0.16
4.84	Acetaldehyde	0.08	0.10	n.d.	-
8.98	Hexanal	5.32	1.51	8.40 *	2.81
14.01	Heptanal	0.36	0.35	0.66 *	0.37
20.93	Nonanal	0.33	0.36	0.55 *	0.30
22.57	2-Furancarboxaldehyde	0.06	0.10	0.17	0.22
23.84	Benzaldehyde	0.18	0.19	0.48 *	0.23
**Total**		**7.59**		11.26 *	
*Ketones*					
2.26	2-Propanone	430.48	86.58	425.12	70.90
3.12	2-Butanone	130.58	14.37	129.04	32.43
4.66	2-Pentanone	0.42	0.21	1.53 *	0.22
6.71	4,4-dimethoxy-2-butanone	0.03	0.06	n.d.	-
13.83	2-Heptanone	0.49	0.41	0.69 *	0.37
14.98	4-Methyl-2-heptanone,	0.03	0.05	0.97 *	0.27
16.30	4,4-Dimethyl-1-penten-3-one	n.d.	-	0.18	0.29
17.67	3-Hydroxy-2-butanone	2.22	1.44	1.47	0.89
17.81	2-Dodecanone	0.05	0.05	0.23	0.14
20.82	2-Nonanone	0.17	0.18	0.18	0.12
28.19	4-Penten-2-one	0.16	0.21	0.25 *	0.20
**Total**		**564.64**		**559.65**	
*Sulphur compounds*					
1.90	Dimethyl sulphide	21.09	6.49	11.02 *	2.80
8.32	Dimethyl disulphide	n.d.	-	0.22	0.26
24.52	Dimethyl sulphoxide	2.57	1.81	2.71	0.94
31.05	Dimethyl sulphone	122.68	61.89	193.41 *	47.08
**Total**		**146.35**		207.36 *	
*Furans*					
15.99	3-methyl-(3H)-isobenzofuran-1-one	0.04	0.04	0.06	0.09
25.93	dihydro-2(3H)-furanone	0.27	0.19	0.30	0.35
29.09	Tetrahydro-6-methyl-2H-pyran-2-one	0.05	0.06	0.17	0.21
**Total**		**0.36**		**0.53**	

Data expressed as ng g^−1^ SI equivalents; * indicates a significant statistical difference compared to raw milk (*p* < 0.05); n.d., not detected.

## Data Availability

The original contributions presented in the study are included in the article/supplementary material, further inquiries can be directed to the corresponding author.

## Supplementary Materials

The following supporting information can be downloaded at: https://www.mdpi.com/article/10.3390/foods13071117/s1, Table S1: VOCs profile of raw non homogenized milk before and after IR treatment with different energies; Table S2: VOC profile of raw homogenized milk before and after IR treatment with energy 80.
