# Supplementary material for: Preliminary Investigation towards the Use of Infrared Technology for Raw Milk Treatment
Source: Foods. 2024 Apr 6;13(7):1117. doi: 10.3390/foods13071117 (PMC11012228; doi:10.3390/foods13071117)
Supplement: Supplementary file 1 [file foods-13-01117-s001.zip › foods-2951110-supplementary.pdf]

Table S1. VOCs profile of raw non homogenized milk before and after IR treatment with different energies.

Rt	Compound	Raw milk (n=3)	s.d. (±)	IR60 (n=3)	s.d. (±)	IR70 (n=3)	s.d. (±)	IR80 (n=3)	s.d. (±)	IR85 (n=3)	s.d. (±)
					Aldehydes						
8.98	Hexanal	0.08	0.16	n.d.	-	0.32	0.63	2.90	1.39	4.21*	1.63
**Total**		**0.08**		**n.d.**		**0.32**		**2.90**		**4.21***	
					Ketones						
2.26	2-Propanone	1212.95	268.77	1405.73	328.22	1369.23*	253.92	1553.42*	472.97	1613.35*	583.46
3.12	2-Butanone	282.75	47.46	268.61	103.14	263.53	67.60	284.33	99.82	282.95	86.80
13.01	2-Heptanone, 6-methyl-	0.26	0.29	0.55	0.29	0.89	0.27	2.93*	2.00	5.01*	3.61
13.83	2-Heptanone	0.49	0.33	0.80	0.80	0.53	0.37	0.33	0.12	0.37	0.22
14.82	Propanone, 1,1-dichloro-	0.19	0.19	0.21	0.11	0.38	0.21	1.05*	0.80	1.77*	1.29
17.67	3-Hydroxy-2-butanone	1.46	1.84	2.08*	2.81	2.02*	2.43	1.06	0.84	0.74	0.24
**Total**		**1498.11**		**1677.98***		**1636.58***		**1843.13***		**1904.19***	
					Sulphur compounds						
1.9	Dimethyl sulphide	48.37	15.09	58.57*	25.59	57.37*	20.13	57.78*	19.86	58.76*	20.86
8.32	Dimethyl disulphide	n.d.	-	0.23	0.27	0.05	0.09	0.33	0.46	0.12	0.11
31.05	Dimethyl sulphone	10.76	6.48	10.08	5.06	14.79	2.94	13.00	4.15	26.40*	11.63
**Total**		**59.13**		**68.88***		**72.21***		**71.11***		**85.28***	
					Carboxylic acids						
22.20	Acetic acid	1.13	0.77	0.79	0.40	1.02	0.42	0.83	0.37	1.10	0.40
26.12	Butanoic acid	11.04	9.59	18.46	16.25	27.15*	9.51	11.61	13.14	19.56	14.38
30.28	Hexanoic acid	4.39	3.43	6.78	5.54	9.07*	2.04	4.88	4.94	7.05	4.28
33.03	Octanoic acid	1.05	0.70	1.60	1.32	1.53	0.61	0.94	0.77	1.16	0.66
35.34	Decanoic acid	0.30	0.20	0.41	0.32	0.40	0.16	0.31	0.26	0.34	0.26
**Total**		**17.90**		**28.05**		**39.17***		**18.57**		**29.21**	
					Alcohols						
1.66	1-Hexanol	5.09	2.85	5.41	2.82	4.27	3.00	6.80	5.06	9.49	6.96
3.14	2-Propanol, 2-methyl-	66.82	79.10	123.35*	59.10	108.67	74.21	104.17	66.32	101.93	49.94
3.67	2-Propanol	2.70	1.66	7.88	11.20	10.72	10.59	2.88	1.41	4.06	2.35
3.77	Ethanol	6.43	1.16	12.34	10.78	13.29	14.53	5.71	1.94	5.77	1.46
16.49	3-Buten-1-ol, 3-methyl-	0.42	0.37	0.59	0.29	0.69	0.44	1.08*	0.80	1.08*	0.59
16.92	1-Pentanol	0.21	0.16	0.25	0.18	0.29	0.26	0.67*	0.27	0.47	0.14
**Total**		**81.67**		**149.83***		**137.93***		**121.31**		**122.79**	
					Ethers						
1.74	Propane, 2-ethoxy-2-methyl-	10.17	8.93	17.40	7.95	16.34	9.87	39.18*	29.22	71.23*	48.06
**Total**		**10.17**		**17.40**		**16.34**		**39.18***		**71.23***	
					Hydrocarbons						
15.11	Cyclopropane, butyl-	0.62	1.00	1.53	1.90	1.43	1.47	0.88	0.33	1.10	0.45

21.66	Benzene, 1,3-bis(1,1-dimethylethyl)-	0.18	0.19	0.25	0.16	0.45	0.10	0.96*	0.53	1.62*	1.10
**Total**		**0.80**		**1.77**		**1.88***		**1.84***		**2.72***	
					Miscellaneous						
28.32	Acetamide	0.03	0.04	n.d.	-	0.05	0.06	0.02	0.04	0.09*	0.06
**Total**		**0.03**		**n.d.**		**0.05**		**0.02**		**0.09***	

Data expressed as ng g^{-1} SI equivalents; * indicate significant statistical difference compared to raw milk ($p<0.05$); n.d., not detected

Table S2. VOC profile of raw homogenized milk before and after IR treatment with energy 80.

Rt	Compound	Raw milk (n=2)	s.d. (±)	IR80 (n=2)	s.d. (±)
		Aldehydes			
3.39	3-Methyl-butanal	1.02	1.09	0.63	0.83
4.74	Pentanal	0.23	0.18	0.37	0.16
4.84	Acetaldehyde	0.08	0.10	n.d.	-
8.98	Hexanal	5.32	1.51	8.40*	2.81
14.01	Heptanal	0.36	0.35	0.66*	0.37
20.93	Nonanal	0.33	0.36	0.55*	0.30
22.57	2-Furancarboxaldehyde	0.06	0.10	0.17	0.22
23.84	Benzaldehyde	0.18	0.19	0.48*	0.23
**Total**		**7.59**		**11.26***	
		Ketones			
2.26	2-Propanone	430.48	86.58	425.12	70.90
3.12	2-Butanone	130.58	14.37	129.04	32.43
4.66	2-Pentanone	0.42	0.21	1.53*	0.22
6.71	4,4-dimethoxy-2-butanone	0.03	0.06	n.d.	-
13.83	2-Heptanone	0.49	0.41	0.69	0.37
14.98	4-Methyl-2-heptanone,	0.03	0.05	0.97*	0.27
16.30	4,4-Dimethyl-1-penten-3-one	n.d.	-	0.18	0.29
17.67	3-Hydroxy-2-butanone	2.22	1.44	1.47	0.89
17.81	2-Dodecanone	0.05	0.05	0.23	0.14
20.82	2-Nonanone	0.17	0.18	0.18	0.12
28.19	4-Penten-2-one	0.16	0.21	0.25*	0.20
**Total**		**564.64**		**559.65**	
		Sulphur compounds			
1.9	Dimethyl sulphide	21.09	6.49	11.02*	2.80
8.32	Dimethyl disulphide	n.d.	-	0.22	0.26
24.52	Dimethyl sulphoxide	2.57	1.81	2.71	0.94
31.05	Dimethyl sulphone	122.68	61.89	193.41*	47.08
**Total**		**146.35**		**207.36***	
		Carboxylic acids			
22.2	Acetic acid	6.70	5.36	4.10	1.90
24.22	Propanoic acid	1.49	0.83	0.94*	0.35
24.89	2-Methyl-propanoic acid	0.23	0.10	0.20	0.08
25.14	2,2-Dimethyl-propanoic acid	0.20	0.13	0.09*	0.10
26.12	Butanoic acid	206.66	141.32	234.77	175.73

26.98	3-Methyl-butanoic acid	0.57	0.58	0.37	0.21
28.28	Pentanoic acid	1.08	0.63	0.88	0.49
30.28	Hexanoic acid	102.76	106.40	110.52	74.59
31.93	Heptanoic acid	0.87	0.66	0.69	0.41
33.03	Octanoic acid	24.51	39.77	11.34	8.64
34.08	Nonanoic acid	0.33	0.24	0.16	0.07
35.34	Decanoic acid	2.27	1.69	1.87	1.42
**Total**		**347.68**		**365.93**	
	Alcohols				
3.67	2-Propanol	0.27	0.17	1.18	1.91
3.77	Ethanol	0.82	0.51	0.65	0.72
12.67	1-Butanol	0.59	0.36	0.73*	0.34
13.87	2-Hexanol	0.63	0.50	0.52	0.32
15.36	3-Methyl-1-Butanol	0.28	0.35	0.06	0.11
16.92	1-Pentanol	0.19	0.15	1.53*	0.64
19.18	1-Hepten-4-ol	0.38	0.24	0.52*	0.33
**Total**		**3.17**		**5.18***	
	Furans				
15.99	3-methyl-(3H)-isobenzofuran-1-one	0.04	0.04	0.06	0.09
25.93	dihydro-2(3H)-furanone	0.27	0.19	0.30	0.35
29.09	Tetrahydro-6-methyl-2H-pyran-2-one	0.05	0.06	0.17	0.21
**Total**		**0.36**		**0.53**	
	Esters				
3.00	Ethyl acetate	0.59	1.18	0.12	0.14
5.02	Butanoic acid, methyl ester	2.39	2.52	1.40	1.75
14.27	Hexanoic acid methyl ester	2.35	2.82	2.06	2.89
15.24	Ethanedioic acid, dibutyl ester	0.01	0.01	0.26	0.30
26.02	Benzoic acid methyl ester	n.d.	-	0.11	0.23
26.74	Phosphoric acid, triethyl ester	0.02	0.03	n.d.	-
**Total**		**5.36**		**3.96**	

Data expressed as ng g^{-1} SI equivalents; * indicate significant statistical difference compared to raw milk ($p<0.05$); n.d., not detected
